# Nonspecific Interstitial Pneumonitis in a Child Associated With Hashimoto Thyroiditis

**DOI:** 10.7759/cureus.26072

**Published:** 2022-06-18

**Authors:** Sinan Yavuz, Doha Mouner, Gangaiah Komala, Ahmed Albarkouky, Mohamed Roshan, Nader Francis

**Affiliations:** 1 Department of Pediatrics, Al Qassimi Women's and Children's Hospital, Sharjah, ARE; 2 Department of Pathology, Al Qassimi Hospital, Sharjah, ARE; 3 Department of Radiology, Al Qassimi Hospital, Sharjah, ARE

**Keywords:** pediatric, childhood interstitial lung disease, hashimoto’s thyroiditis, pediatrics, non-specific interstitial lung disease (nsip)

## Abstract

Interstitial lung disease (ILD) is a rare disease defined as a specific type of chronic fibrosing interstitial pneumonitis whose effects are limited to the lung. Nonspecific interstitial pneumonia (NSIP) was defined as a histopathological form that can be seen in the presence of large different clinical and radiological features.

The exact role of thyroid hormone in the pathogenetic mechanism of idiopathic interstitial pneumonitis (IIP) is unclear. But there is a suggestion that the thyroid hormone plays a role in pulmonary inflammation and fibrosis. In this case report, we described the presentation of NSIP which was associated with Hashimoto thyroiditis.

## Introduction

Children’s interstitial lung disease (chILD) is a rare group that consists of more than 200 conditions and they are chronic progressive diseases. Childhood interstitial pneumonitis (IP) has a broad spectrum of histologic anomalies that do not meet the criteria of adult IP classification [1-3]. The definition of nonspecific interstitial pneumonitis (NSIP) is a histopathological way that can be seen in large differentiation of radiological and clinical presentation [4]. NSIP is defined as “idiopathic” [5] or “secondary” in view of the absence or presence of the etiological cause. The “clinical diagnosis” of NSIP is kept for idiopathic and biopsy-proven cases only [4,6].

Although NSIP is seen in many conditions like connective tissue disorders, reactions to certain medications, HIV, hypersensitivity pneumonitis, and infection, however, most of the cases are idiopathic [4]. The exact role of thyroid hormone in the pathogenesis of idiopathic interstitial pneumonitis (IIP) is unknown. But there is a suggestion that the thyroid hormone plays a role in pulmonary inflammation and fibrosis. Sato et al. have shown the link between abnormal thyroid function and/or positive thyroid antibody and IIP with autoimmune features [7].

Here, we present a case report of a child with infantile spasm, Hashimoto thyroiditis, and recurrent respiratory infections during the first five years of life. Her whole-genome sequencing (WES) showed heterozygous duplication of exons 1-3 GNAS Chr 20q 13.32. Her clinical and radiological findings were going with NSIP, and her diagnosis was confirmed histopathologically.

## Case presentation

A five-year-old girl presented with a history of high-grade fever, productive cough, and shortness of breath for five days. She had recurrent admission due to a chest infection in her past medical history. She was diagnosed with infantile spasms at the age of six months and started on the epileptic drug. During the treatment for infantile spasms, she received prednisolone, and at that time, she did not complain of chest issues. When she was two years old, she was diagnosed with asthma and started on a prophylactic inhaled steroid (Fluticasone propionate inhaler). Also, she was receiving intravenous or oral steroids at 1 mg/kg/day for five days each admission. At the age of five years, she was diagnosed with Hashimoto thyroiditis. At the time of admission to the hospital, physical examination revealed an alert child, with no distinctive features apart from mild bilateral ptosis, fingers clubbing, and growth parameters within the 25th to 50th centile. Chest examination showed good air entry bilaterally with scattered fine crackles and wheezes and mild tachypnea. Other examinations were unremarkable.

**Figure 1:**
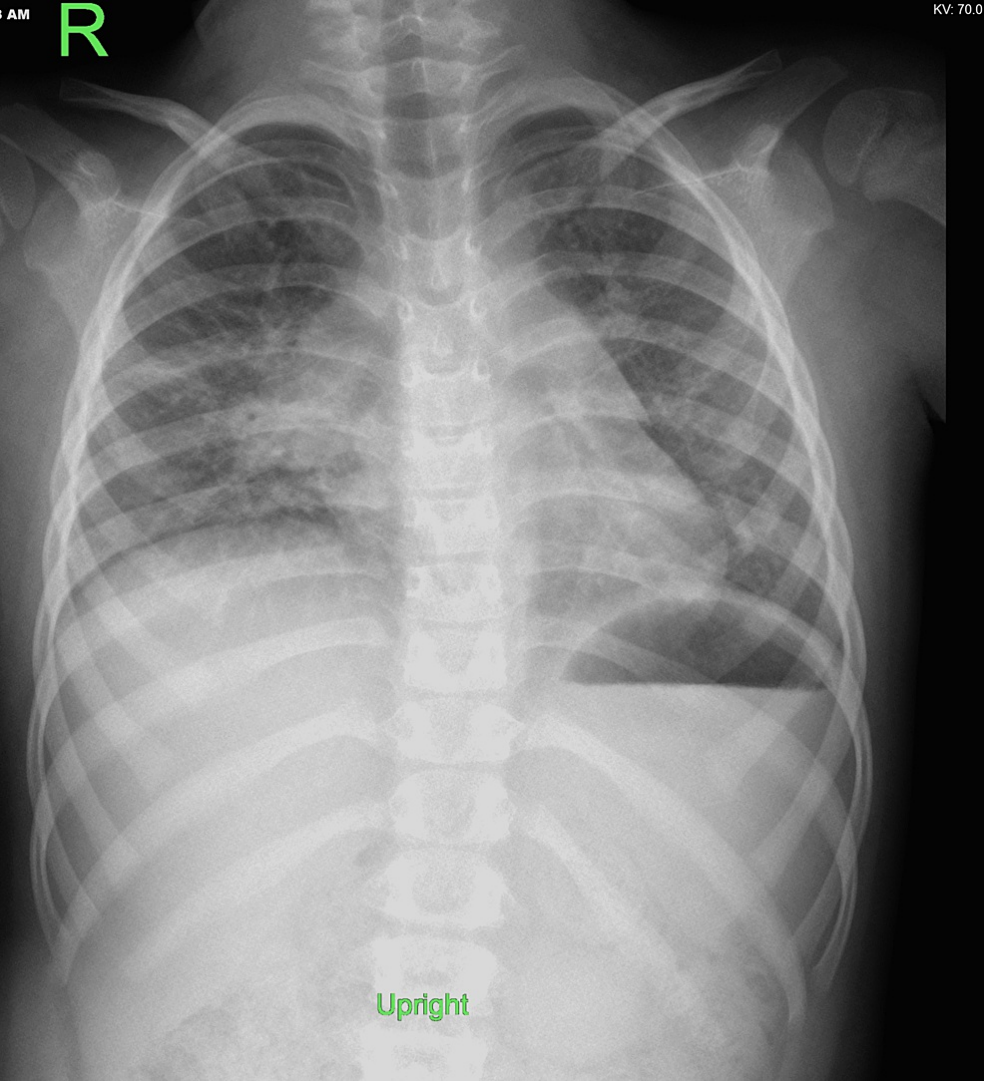
Chest X-ray on admission showed right lung midzone and left retrocardiac lower and mid-zone air space opacity with air bronchograms.

Complete blood count (CBC), C-reactive protein (CRP), electrolyte, and liver function tests were normal. Chest X-ray showed right lung midzone and left retrocardiac lower and mid-zone air space opacity with air bronchograms (Figure 1).

**Figure 2:**
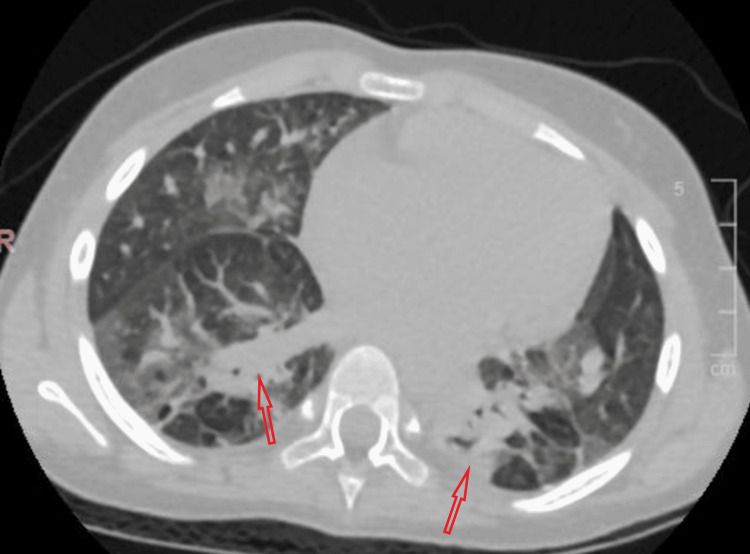
CT chest showed bilateral consolidation (red arrows) with air bronchogram.

**Figure 3:**
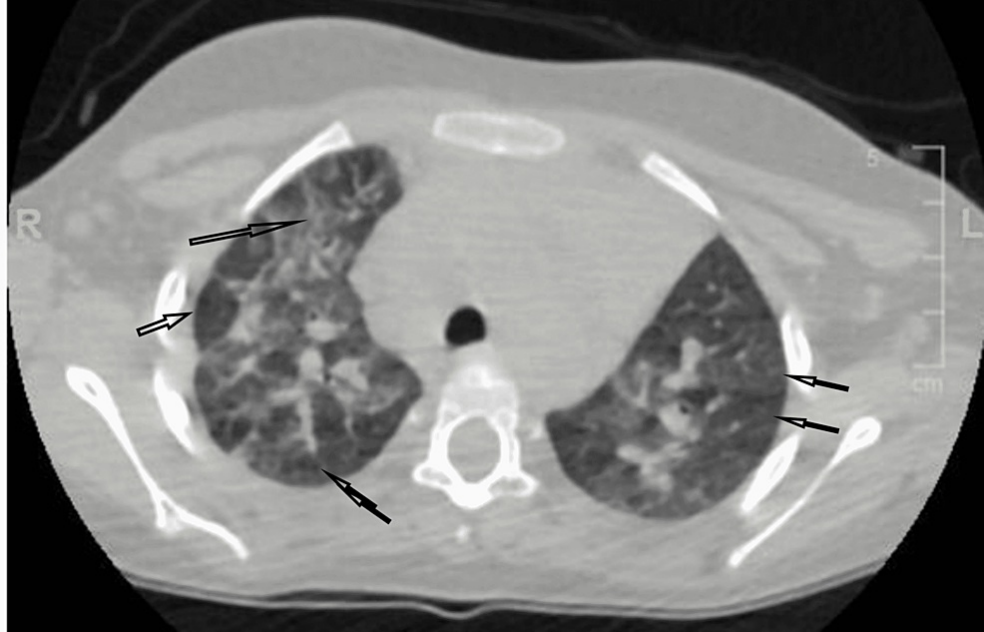
CT chest showed bilateral multiple patchy areas of differential pulmonary attenuation (mosaic pattern of lung attenuation) (black arrows).

CT chest showed bilateral consolidation with air bronchograms and predominance towards hilar and basal segments, bilateral multiple patchy areas of differential pulmonary attenuation (mosaic pattern of lung attenuation) (Figures 2, 3).

Because of the clinical and radiological features suggesting ILD, we decided to extend our study. Antinuclear antibody (ANA), anti-DNA, antineutrophil cytoplasmic antibody (ANCA), perinuclear anti-neutrophil cytoplasmic antibodies (P-ANCA), immunoglobulins, urine organic acids, PCR-HIV, glutamic acid decarboxylase (GAD), anti-tissue transglutaminase (TTG), upper gastrointestinal (GI) study, MRI scan, MicroArray, and fluorescence in situ hybridization (FISH) were normal. WES showed heterozygous duplication of exons 1-3 GNAS Chr 20q 13.32. Thyroid-stimulating hormone (TSH) and thyroid peroxidase antibodies (TPO-Ab) were high. A bronchoscopy showed normal anatomy and purulent secretion, mainly from the right lung. Bronchoalveolar lavage (BAL) histopathology revealed chronic inflammation, and BAL culture was negative. Blood and lavage fluids were not showing any evidence suggesting a tumor.

**Figure 4:**
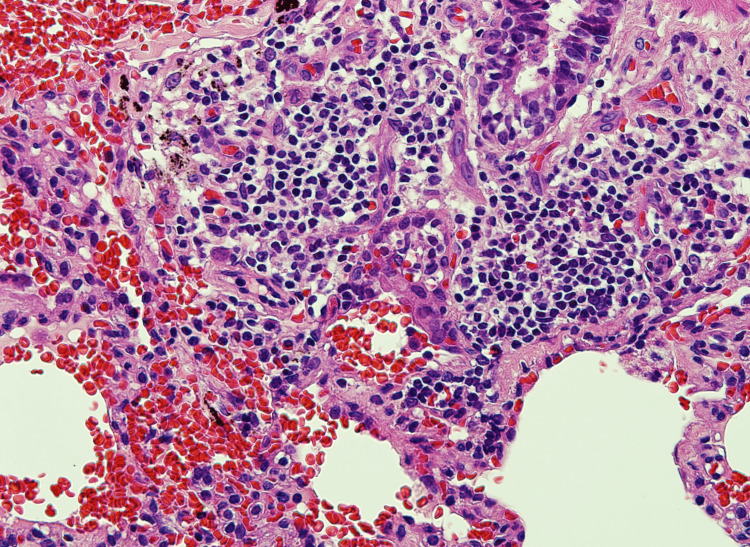
Lung biopsy revealed the bronchial wall has moderate aggregates of small lymphocytes.

**Figure 5:**
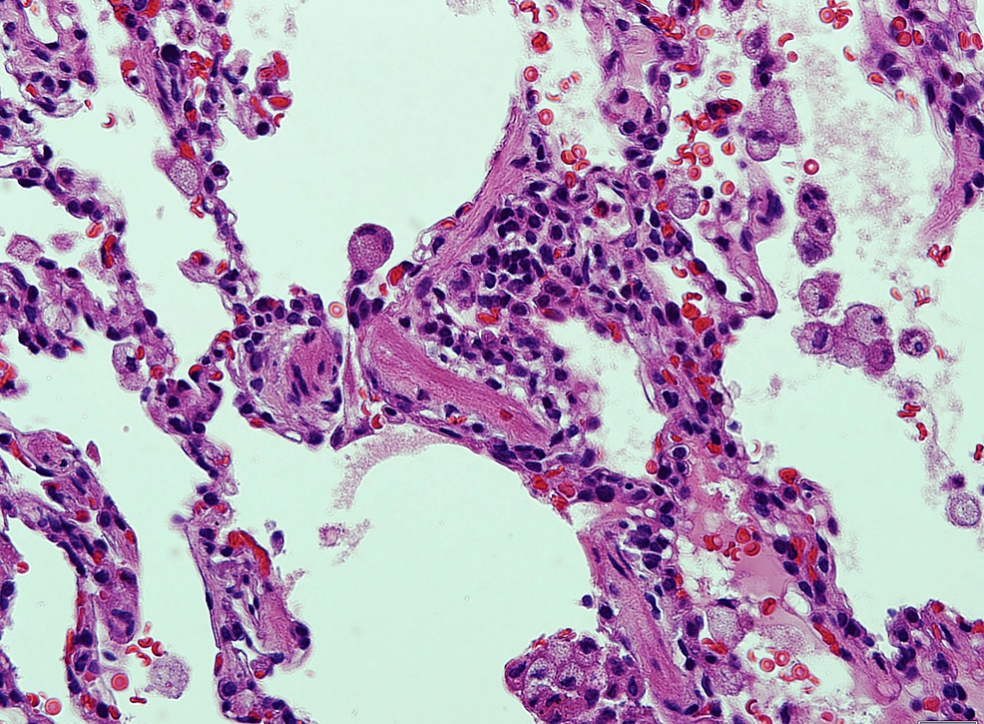
Lung biopsy revealed alveolar spaces of variable sizes with mild cellularity of their wall by alveolar macrophages and small lymphocytes.

**Figure 6:**
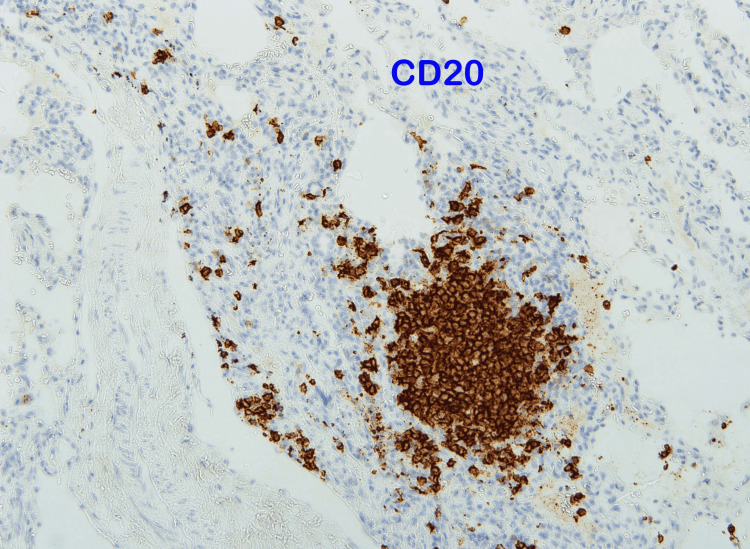
Lung biopsy showed positive for B-lymphocytes at the bronchiolar wall and few in the alveolar wall.

**Figure 7:**
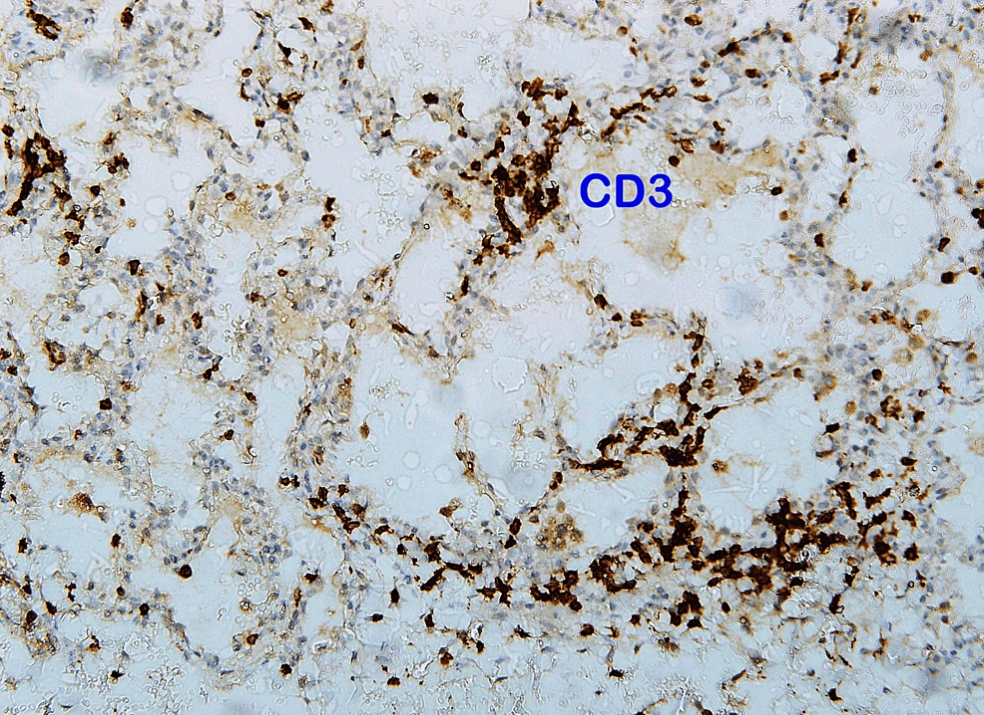
Lung biopsy showed positive for T-lymphocytes at the alveolar wall.

The findings were in favor of interstitial lung disease. For the final diagnosis, a lung biopsy was done. Histopathology materials and methods were as follows: paraffin sections were stained by routine stain - hematoxylin and eosin (H&E) stain; immunohistochemistry (IHC) using DAKO stains by full automated DAKO immune-stainer; CD20 as a marker for B-lymphocytes and CD3 as a marker for T-lymphocytes were used. It showed lung parenchyma having maintained architecture with moderate small lymphocytic infiltration in the bronchiolar wall and mild to moderate in the alveolar wall that was consistent with chronic bronchiolitis with mild to moderate nonspecific interstitial pneumonitis pattern. In favor of hypersensitivity reaction with lymphocytic interstitial pneumonitis. T- lymphocytes admixed with B-lymphocytes in the inflammatory reaction and are more pronounced in the alveolar wall (Figures 4-7).

**Figure 8:**
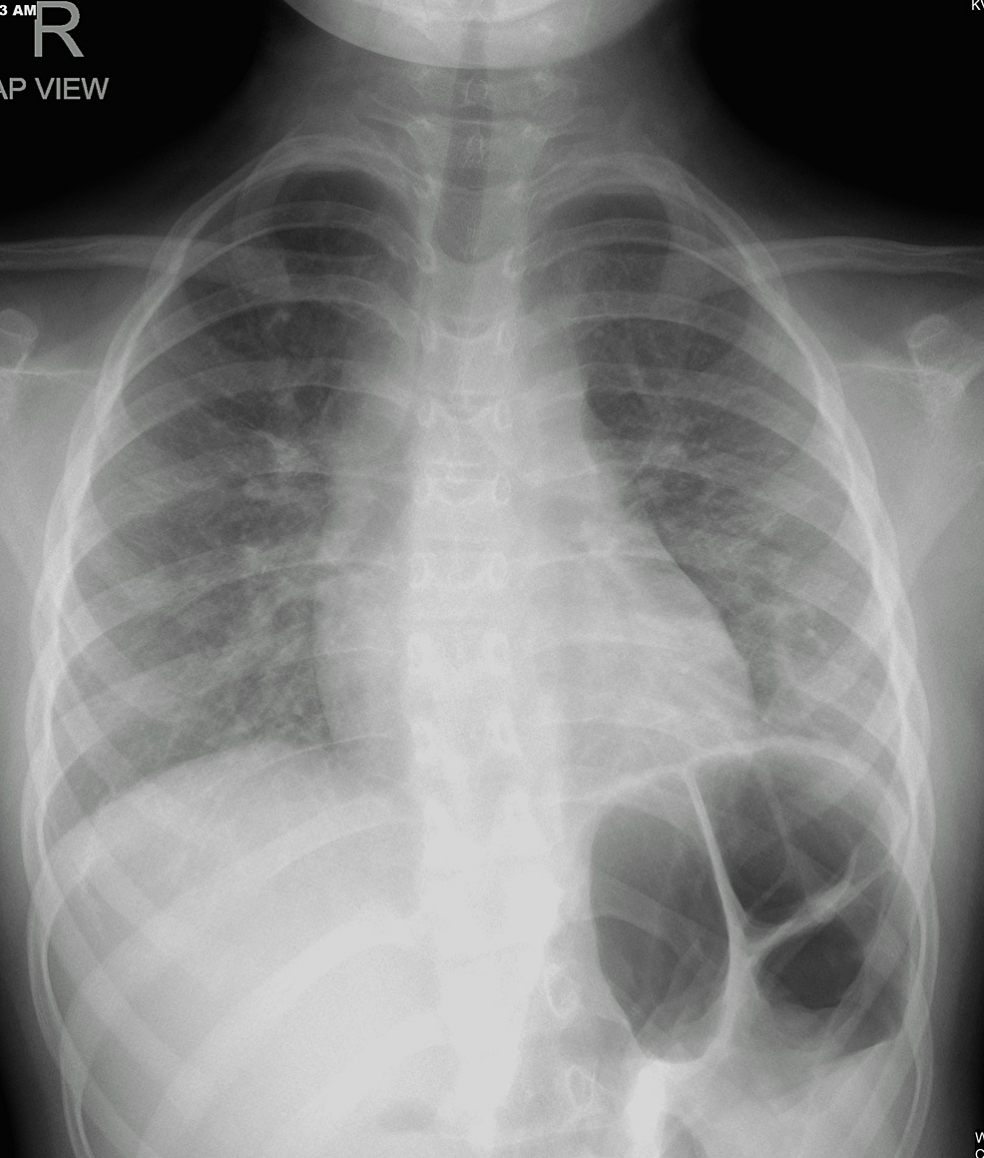
A follow-up chest X-ray showed significant improvement compared to the previous one.

The diagnosis of NSIP was made based on clinical, radiological, and histopathological findings and was associated with Hashimoto thyroiditis. Oral prednisolone was administered at 1 mg/kg/day for one year. A follow-up chest X-ray showed significant improvement compared to the previous one (Figure 8).

Over the two-year post-treatment follow-up, she showed a significant clinical improvement in terms of no more hospital admission and no relapse.

## Discussion

Like the adult type, nonspecific interstitial pneumonia in children is a subtype of ILD that has a better prognosis than idiopathic pulmonary fibrosis/usual interstitial pneumonia [8]. The diagnosis was made on clinical examination, imaging, and histopathology findings. The signs and symptoms are dry cough, shortness of breath during exertion that worsens over time, difficult or labored breathing, fatigue, and clubbing [4]. Our patient had typical clinical signs of NSIP cellular type, like the presence of clubbing at presentation and late inspiratory crackles.

In IIP, inflammation and fibrosis in the lung develop due to extreme oxidants, which is thyroid hormone playing an important role. Also, they have a prevention role by controlling the function and state of developing macrophages as well as reducing fibrotic change by stopping TGF-β signaling and enhancing epithelial mitochondrial role [9-14]. Recently a study done in the adult population has revealed, in patients with IIP and non-idiopathic pulmonary fibrosis (IPF), a positive effect between free T3 (tri-iodothyronine) (FT3) and vital capacity and vice versa with inflammatory markers like CRP and erythrocyte sedimentation rate (ESR) [7]. There is a lack of data in children about the relation between Hashimoto thyroiditis and NSIP, as most cases were reported in adults. Unfortunately, the diagnosis made at the age of five years could be a late diagnosis in our case. We hypothesize that NSIP is secondary to Hashimoto thyroiditis, but we need more data in children to describe all presentations.

The common radiological findings consist of symmetric and diffuse ground-glass opacities, bilateral (86%) or basal predominance, subpleural sparing (relatively specific sign), reticular opacities, irregular linear opacities, subpleural reticulation, thickening of bronchovascular bundles, traction bronchiectasis. The distribution of ground-glass opacity or consolidation is typically peripheral or subpleural spaces [15]. In the present case, high-resolution computed tomography (HRCT) showed differences from typical radiological features of cellular NSIP (c-NSIP). The patient had lobar and multilobar consolidation, which is not the typical feature of NSIP. Ground glass opacities were absent in our case, which is commonly seen in most cases of NSIP.

NSIP is divided into two subtypes: cellular and fibrotic. The characterization of the cellular form is inflammation of the cells of the interstitium, and the fibrotic form is thickening and irreversible fibrotic changes [15]. The histological findings of c-NSIP consist of mild to moderate interstitial chronic inflammation and type II pneumocyte hyperplasia in areas of inflammation. In comparison, the fibrosing type of NSIP findings is dense or loose interstitial fibrosis that lacks the temporal heterogeneity pattern [16]. In cellular type, the cornerstone of treatment is a corticosteroid. NSIP has the best outcome compared with other idiopathic interstitial pneumonia [17]. The same happened with our patient, and she improved dramatically after starting the treatment with the corticosteroid.

Regarding the WES result, there is no clear data on whether there is any correlation between the gene and lung disease.

## Conclusions

There are few published studies of NSIP associated with Hashimoto thyroiditis in pediatrics, especially those below five years, as most cases have been published in the adult age group. In this case report, a case of Hashimoto thyroiditis was associated with NSIP. There is no evidence of an association between infantile spasm and NSIP, and it is considered a co-accident finding. Our patient was started on an oral corticosteroid that dramatically improved her symptoms and quality of life with no more hospital admission.
